# Ethyl 7-methyl-2-((1-methyl-1*H*-pyrrol-2-yl)methyl­ene)-3-oxo-5-phenyl-3,5-dihydro-2*H*-thia­zolo[3,2-*a*]pyrimidine-6-carboxyl­ate

**DOI:** 10.1107/S1600536812041748

**Published:** 2012-10-10

**Authors:** Jie Hu, Xi-Xi Wu, Xue-Qian Shen, Long-Guang Tang, Xiao-Kun Li

**Affiliations:** a Wenzhou Medical College, School of Pharmacy, Wenzhou 325035, People’s Republic of China

## Abstract

In the structure of the title compound, C_22_H_21_N_3_O_3_S, the thia­zole ring forms dihedral angles of 88.83 (7) and 9.39 (9)°, respectively, with the benzene and pyrrole rings. The dihydro­pyrimidine ring adopts a flattened boat conformation. The olefinic double bond is in a *Z* conformation.

## Related literature

For related structures, see: Hou (2009 ▶); Zhao *et al.* (2011 ▶). For background to the biological properties of fused thia­zolo[3,2-*a*]pyrimidine derivatives, see: Ashok *et al.* (2007 ▶); Bahekar & Shinde (2004 ▶); Hurst & Hull (1961 ▶); Mehta *et al.* (2006 ▶); Shah & Desai (2007 ▶); Srivastava *et al.* (2006 ▶); Subudhi *et al.* (2007 ▶); Magerramov *et al.* (2006 ▶); Zhou *et al.* (2008 ▶).
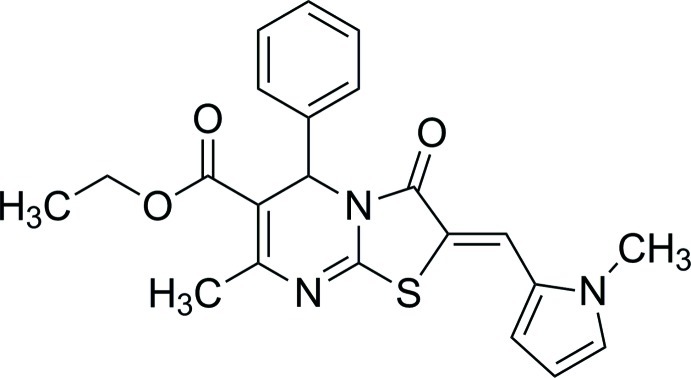


## Experimental

### 

#### Crystal data

C_22_H_21_N_3_O_3_S
*M* *_r_* = 407.48
Monoclinic, 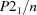

*a* = 11.8187 (10) Å
*b* = 10.2911 (9) Å
*c* = 16.2290 (14) Å
β = 90.584 (2)°
*V* = 1973.8 (3) Å^3^
*Z* = 4
Mo *K*α radiation
μ = 0.19 mm^−1^
*T* = 293 K
0.32 × 0.24 × 0.16 mm

#### Data collection

Bruker SMART CCD area-detector diffractometer
Absorption correction: multi-scan (*SADABS*; Bruker, 2002 ▶) *T*_min_ = 0.814, *T*_max_ = 1.000
10415 measured reflections
3877 independent reflections
3433 reflections with *I* > 2σ(*I*)
*R*_int_ = 0.020

#### Refinement

*R*[*F* ^2^ > 2σ(*F* ^2^)] = 0.040
*wR*(*F* ^2^) = 0.109
*S* = 1.05
3877 reflections
265 parameters
H-atom parameters constrained
Δρ_max_ = 0.30 e Å^−3^
Δρ_min_ = −0.19 e Å^−3^

### 

Data collection: *SMART* (Bruker, 2002 ▶); cell refinement: *SAINT* (Bruker, 2002 ▶); data reduction: *SAINT*; program(s) used to solve structure: *SHELXS97* (Sheldrick, 2008 ▶); program(s) used to refine structure: *SHELXL97* (Sheldrick, 2008 ▶); molecular graphics: *SHELXTL* (Sheldrick, 2008 ▶); software used to prepare material for publication: *SHELXTL*.

## Supplementary Material

Crystal structure: contains datablock(s) I, New_Global_Publ_Block. DOI: 10.1107/S1600536812041748/aa2075sup1.cif

Structure factors: contains datablock(s) I. DOI: 10.1107/S1600536812041748/aa2075Isup2.hkl

Additional supplementary materials:  crystallographic information; 3D view; checkCIF report

## Figures and Tables

**Fig. 1.**
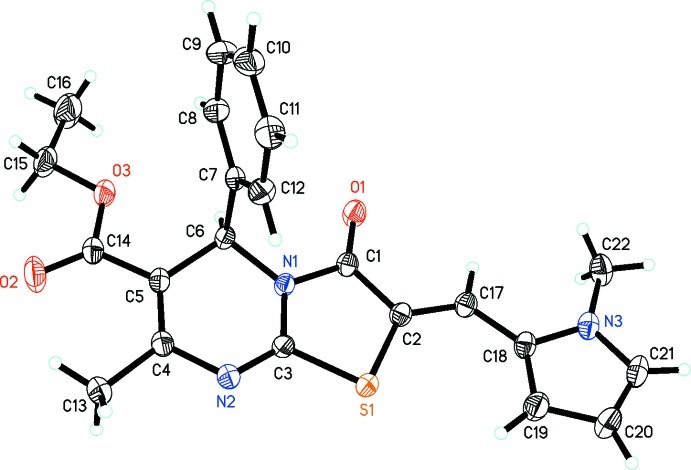
The molecular structure of the title compound, showing 30% displacement ellipsoids for the non-hydrogen atoms. Hydrogen atoms are drawn as spheres of arbitrary radius.

## supplementary crystallographic information

### Comment

Thiazolinone and their derivatives have attracted continuing interest over the years because of their varied biological activities (Shah & Desai,2007), such as antifungal (Mehta *et al.*, 2006), antibacterial (Subudhi *et al.*, 2007), anti-tumor (Zhou *et al.*, 2008), anti-HIV and anti-inflammatory (Srivastava *et al.*, 2006). 3,4-Dihydropyrimidin-2(1*H*)-ones (DHPMs) are known for more than a century and have attracted considerable attention because of their wide spectrum of therapeutic and pharmacological properties. DHPMs have been used as antibacterial, antifungal (Ashok *et al.*, 2007), antiviral (Hurst & Hull, 1961), anti-inflammatory (Bahekar & Shinde, 2004), antioxidative properties and noteworthy, as well as calcium channel modulators (Magerramov *et al.*, 2006). Herein, we report in the present work based on the pharmacological principle of stacking, such biologically active groups as DHPMs was introduced to thiazolinone, with a view to get new compounds with better bioactivity.

In continuation of our studies on heterocyclic compounds, we report the crystal structure of the title compound. The fused thiazole ring has usual geometry as observed in other thiazolo[3,2-*a*]pyrimidine compounds (Hou, 2009; Zhao *et al.*, 2011). The thiazole ring makes dihedral angles of 88.83 (7) and 9.39 (9)° with the benzene ring and pyrrole ring, respectively. The pyrimidine ring adopts a flattened boat conformation. The C2–C17 distance, 1.345 (2) Å, confirms this as a double bond and the molecule adopts a *Z* conformation with respect to this bond (Fig. 1).

### Experimental

In a typical procedure of one pot Biginelli reaction, sulfamic acid (0.4 mol) was added to a solution of substituted benzaldehyde (0.5 mol), ethyl acetylacetate (0.6 mol), and thiourea (0.75 mol) in ethanol and reflux at 351 K for 2 h. When the reaction was finished, the mixture was cooled to room temperature and filtered. The product ethyl 2-mercapto-4-methyl-6 -phenyl-1,6-dihydropyrimidine-5-carboxylate was washed with water, and then dried in vacuum as a white solid.

To a stirred solution of ethyl ] 2-mercapto-4-methyl-6-phenyl-1,6-dihydropyrimidine-5-carboxylate (2 mmol) and ethyl chloroacetate (2 mmol) in ethanol (10 ml) pyridine (2 mmol) was added.The reaction was heated at refluxing temperature for 4 h. Then 1-methyl-1*H*-pyrrole-2-carbaldehyde (2 mmol) and morpholine (2 mmol) was added to the mixture without further treatment until the reaction finished. The mixture was then cooled to room temperature, filtered and washed with water to obtain crude product. The resulting yellow solid was collected and recrystallized from acetic acid, then single crystals were grown in CH_2_Cl_2_/CH_3_OH mixture (2:1). Yield 45.6%.

^1^H NMR (DMSO-d_6_) δ: 1.111 (3*H*, m, 6–CH_3_), 4.030 (2*H*, m, 6–CH_2_), 2.380 (3*H*, s, N–CH_3_), 3.730 (3*H*, s, 7–CH_3_), 6.035 (H, s, 5–CH), 6.317 (1*H*, m, pyrrole), 6.576(1*H*, m, pyrrole), 7.213 (1*H*, m, pyrrole), 7.284–7.340 (5*H*, m, Ar—H), 7.625 (1*H*, s, =CH). ESI-MS m/*z*: 408.4 (*M*)^+^, 430.3 (*M*+Na)^+^, calcd for C_22_H_21_N_3_O_3_S 407.49.

### Refinement

The H atoms were positioned geometrically (C—H = 0.93 – 0.98 Å) and refined as riding with *U*_iso_(H) = 1.2*U*_eq_(C) or 1.5*U*_eq_(methyl C).

### Crystal data

**Table:**

C_22_H_21_N_3_O_3_S	*F*(000) = 856
*M**_r_* = 407.48	*D*_x_ = 1.371 Mg m^−^^3^
Monoclinic, *P*2_1_/*n*	Mo *K*α radiation, λ = 0.71073 Å
Hall symbol: -P 2yn	Cell parameters from 4867 reflections
*a* = 11.8187 (10) Å	θ = 5.0–56.3°
*b* = 10.2911 (9) Å	µ = 0.19 mm^−^^1^
*c* = 16.2290 (14) Å	*T* = 293 K
β = 90.584 (2)°	Prismatic, red
*V* = 1973.8 (3) Å^3^	0.32 × 0.24 × 0.16 mm
*Z* = 4	

### Data collection

**Table:**

Bruker SMART CCD area-detector diffractometer	3877 independent reflections
Radiation source: fine-focus sealed tube	3433 reflections with *I* > 2σ(*I*)
Graphite monochromator	*R*_int_ = 0.020
phi and ω scans	θ_max_ = 26.0°, θ_min_ = 2.1°
Absorption correction: multi-scan (*SADABS*; Bruker, 2002)	*h* = −14→14
*T*_min_ = 0.814, *T*_max_ = 1.000	*k* = −12→11
10415 measured reflections	*l* = −20→19

### Refinement

**Table:**

Refinement on *F*^2^	Primary atom site location: structure-invariant direct methods
Least-squares matrix: full	Secondary atom site location: difference Fourier map
*R*[*F*^2^ > 2σ(*F*^2^)] = 0.040	Hydrogen site location: inferred from neighbouring sites
*wR*(*F*^2^) = 0.109	H-atom parameters constrained
*S* = 1.05	*w* = 1/[σ^2^(*F*_o_^2^) + (0.0574*P*)^2^ + 0.5784*P*] where *P* = (*F*_o_^2^ + 2*F*_c_^2^)/3
3877 reflections	(Δ/σ)_max_ < 0.001
265 parameters	Δρ_max_ = 0.30 e Å^−^^3^
0 restraints	Δρ_min_ = −0.19 e Å^−^^3^

### Special details

**Table:**

Geometry. All e.s.d.'s (except the e.s.d. in the dihedral angle between two l.s. planes) are estimated using the full covariance matrix. The cell e.s.d.'s are taken into account individually in the estimation of e.s.d.'s in distances, angles and torsion angles; correlations between e.s.d.'s in cell parameters are only used when they are defined by crystal symmetry. An approximate (isotropic) treatment of cell e.s.d.'s is used for estimating e.s.d.'s involving l.s. planes.
Refinement. Refinement of *F*^2^ against ALL reflections. The weighted *R*-factor *wR* and goodness of fit *S* are based on *F*^2^, conventional *R*-factors *R* are based on *F*, with *F* set to zero for negative *F*^2^. The threshold expression of *F*^2^ > σ(*F*^2^) is used only for calculating *R*-factors(gt) *etc*. and is not relevant to the choice of reflections for refinement. *R*-factors based on *F*^2^ are statistically about twice as large as those based on *F*, and *R*- factors based on ALL data will be even larger.

### Fractional atomic coordinates and isotropic or equivalent isotropic displacement parameters (Å^2^)

**Table:**

	*x*	*y*	*z*	*U*_iso_*/*U*_eq_	
S1	0.45859 (3)	0.25287 (4)	0.01131 (2)	0.04128 (14)	
N1	0.36364 (10)	0.46192 (12)	0.06560 (7)	0.0318 (3)	
N2	0.26786 (11)	0.35881 (14)	−0.04481 (8)	0.0402 (3)	
N3	0.76832 (11)	0.15037 (14)	0.18041 (8)	0.0413 (3)	
O1	0.48083 (10)	0.52724 (11)	0.17107 (8)	0.0471 (3)	
O2	0.05024 (13)	0.69653 (15)	−0.05111 (10)	0.0735 (5)	
O3	0.14144 (10)	0.76092 (11)	0.06154 (7)	0.0473 (3)	
C1	0.45910 (12)	0.45135 (15)	0.11618 (9)	0.0337 (3)	
C2	0.52334 (12)	0.33419 (15)	0.09404 (9)	0.0343 (3)	
C3	0.34879 (12)	0.36725 (15)	0.00763 (9)	0.0338 (3)	
C4	0.19135 (13)	0.46391 (15)	−0.04784 (9)	0.0362 (3)	
C5	0.19370 (12)	0.56033 (15)	0.00841 (9)	0.0336 (3)	
C6	0.27014 (12)	0.55164 (14)	0.08448 (9)	0.0316 (3)	
H6	0.3014	0.6379	0.0965	0.038*	
C7	0.20515 (11)	0.50354 (15)	0.15961 (9)	0.0330 (3)	
C8	0.16948 (14)	0.59098 (18)	0.21843 (10)	0.0446 (4)	
H8	0.1883	0.6784	0.2138	0.054*	
C9	0.10566 (17)	0.5486 (2)	0.28426 (11)	0.0588 (5)	
H9	0.0809	0.6081	0.3233	0.071*	
C10	0.07856 (16)	0.4197 (2)	0.29245 (11)	0.0573 (5)	
H10	0.0360	0.3919	0.3370	0.069*	
C11	0.11433 (15)	0.3322 (2)	0.23496 (11)	0.0520 (4)	
H11	0.0967	0.2446	0.2406	0.062*	
C12	0.17686 (14)	0.37389 (17)	0.16826 (10)	0.0422 (4)	
H12	0.2001	0.3141	0.1289	0.051*	
C13	0.11228 (15)	0.45209 (19)	−0.12025 (11)	0.0485 (4)	
H13A	0.0502	0.5112	−0.1137	0.073*	
H13B	0.0840	0.3648	−0.1235	0.073*	
H13C	0.1520	0.4728	−0.1699	0.073*	
C14	0.12023 (13)	0.67570 (16)	0.00121 (10)	0.0396 (4)	
C15	0.07470 (16)	0.87886 (19)	0.05991 (13)	0.0561 (5)	
H15A	−0.0034	0.8591	0.0727	0.067*	
H15B	0.0768	0.9173	0.0054	0.067*	
C16	0.1209 (2)	0.9705 (3)	0.12081 (18)	0.0907 (9)	
H16A	0.1136	0.9345	0.1751	0.136*	
H16B	0.0799	1.0509	0.1176	0.136*	
H16C	0.1993	0.9861	0.1096	0.136*	
C17	0.61539 (12)	0.29575 (16)	0.13690 (10)	0.0371 (3)	
H17	0.6411	0.3529	0.1773	0.045*	
C18	0.67851 (12)	0.17881 (16)	0.12828 (10)	0.0384 (4)	
C19	0.66600 (15)	0.07268 (18)	0.07624 (12)	0.0503 (4)	
H19	0.6123	0.0643	0.0343	0.060*	
C20	0.74750 (17)	−0.01876 (19)	0.09753 (13)	0.0572 (5)	
H20	0.7581	−0.0994	0.0729	0.069*	
C21	0.80908 (15)	0.03176 (18)	0.16125 (12)	0.0516 (4)	
H21	0.8697	−0.0091	0.1875	0.062*	
C22	0.81581 (16)	0.23339 (19)	0.24420 (13)	0.0548 (5)	
H22A	0.8705	0.1853	0.2760	0.082*	
H22B	0.7565	0.2628	0.2796	0.082*	
H22C	0.8518	0.3069	0.2192	0.082*	

### Atomic displacement parameters (Å^2^)

**Table:**

	*U*^11^	*U*^22^	*U*^33^	*U*^12^	*U*^13^	*U*^23^
S1	0.0416 (2)	0.0415 (2)	0.0407 (2)	0.01238 (17)	−0.00433 (17)	−0.00727 (16)
N1	0.0271 (6)	0.0325 (7)	0.0358 (6)	0.0032 (5)	−0.0009 (5)	−0.0010 (5)
N2	0.0418 (7)	0.0412 (8)	0.0373 (7)	0.0070 (6)	−0.0067 (6)	−0.0036 (6)
N3	0.0334 (7)	0.0417 (8)	0.0488 (8)	0.0044 (6)	−0.0002 (6)	0.0088 (6)
O1	0.0408 (6)	0.0430 (7)	0.0573 (7)	0.0047 (5)	−0.0145 (5)	−0.0128 (6)
O2	0.0766 (10)	0.0619 (9)	0.0813 (10)	0.0293 (8)	−0.0421 (8)	−0.0117 (8)
O3	0.0500 (7)	0.0418 (7)	0.0499 (7)	0.0171 (5)	−0.0098 (5)	−0.0021 (5)
C1	0.0281 (7)	0.0335 (8)	0.0396 (8)	−0.0014 (6)	−0.0007 (6)	0.0012 (6)
C2	0.0296 (7)	0.0346 (8)	0.0388 (8)	0.0004 (6)	0.0019 (6)	0.0010 (6)
C3	0.0346 (7)	0.0338 (8)	0.0331 (7)	0.0041 (6)	0.0019 (6)	0.0005 (6)
C4	0.0334 (7)	0.0393 (8)	0.0359 (7)	0.0015 (6)	−0.0023 (6)	0.0039 (6)
C5	0.0289 (7)	0.0357 (8)	0.0363 (7)	0.0019 (6)	−0.0008 (6)	0.0055 (6)
C6	0.0283 (7)	0.0288 (7)	0.0376 (8)	0.0036 (6)	−0.0016 (6)	−0.0014 (6)
C7	0.0263 (7)	0.0398 (8)	0.0328 (7)	0.0047 (6)	−0.0051 (6)	−0.0007 (6)
C8	0.0474 (9)	0.0451 (10)	0.0413 (9)	0.0062 (8)	−0.0028 (7)	−0.0075 (7)
C9	0.0566 (11)	0.0806 (15)	0.0395 (9)	0.0131 (10)	0.0064 (8)	−0.0122 (9)
C10	0.0463 (10)	0.0845 (16)	0.0412 (9)	0.0007 (10)	0.0076 (8)	0.0094 (9)
C11	0.0436 (9)	0.0572 (11)	0.0554 (10)	−0.0039 (8)	0.0033 (8)	0.0114 (9)
C12	0.0403 (8)	0.0430 (9)	0.0433 (9)	0.0009 (7)	0.0033 (7)	−0.0006 (7)
C13	0.0467 (9)	0.0545 (11)	0.0442 (9)	0.0063 (8)	−0.0121 (7)	−0.0035 (8)
C14	0.0358 (8)	0.0402 (9)	0.0427 (8)	0.0044 (7)	−0.0033 (7)	0.0062 (7)
C15	0.0539 (11)	0.0439 (10)	0.0703 (12)	0.0189 (8)	−0.0066 (9)	−0.0017 (9)
C16	0.0925 (18)	0.0751 (17)	0.1037 (19)	0.0376 (14)	−0.0330 (15)	−0.0384 (14)
C17	0.0305 (7)	0.0371 (8)	0.0437 (8)	0.0001 (6)	−0.0004 (6)	0.0003 (7)
C18	0.0294 (7)	0.0393 (9)	0.0465 (9)	0.0029 (6)	0.0007 (6)	0.0054 (7)
C19	0.0444 (9)	0.0479 (10)	0.0584 (11)	0.0083 (8)	−0.0045 (8)	−0.0052 (8)
C20	0.0566 (11)	0.0425 (10)	0.0725 (13)	0.0140 (9)	0.0016 (10)	−0.0040 (9)
C21	0.0438 (9)	0.0447 (10)	0.0663 (12)	0.0142 (8)	0.0024 (8)	0.0129 (9)
C22	0.0479 (10)	0.0532 (11)	0.0628 (12)	0.0022 (8)	−0.0167 (9)	0.0072 (9)

### Geometric parameters (Å, º)

**Table:**

S1—C2	1.7515 (15)	C9—H9	0.9300
S1—C3	1.7525 (15)	C10—C11	1.367 (3)
N1—C3	1.3644 (19)	C10—H10	0.9300
N1—C1	1.3927 (18)	C11—C12	1.385 (2)
N1—C6	1.4747 (18)	C11—H11	0.9300
N2—C3	1.2767 (19)	C12—H12	0.9300
N2—C4	1.410 (2)	C13—H13A	0.9600
N3—C21	1.350 (2)	C13—H13B	0.9600
N3—C18	1.382 (2)	C13—H13C	0.9600
N3—C22	1.451 (2)	C15—C16	1.467 (3)
O1—C1	1.2103 (18)	C15—H15A	0.9700
O2—C14	1.1986 (19)	C15—H15B	0.9700
O3—C14	1.336 (2)	C16—H16A	0.9600
O3—C15	1.448 (2)	C16—H16B	0.9600
C1—C2	1.471 (2)	C16—H16C	0.9600
C2—C17	1.345 (2)	C17—C18	1.424 (2)
C4—C5	1.348 (2)	C17—H17	0.9300
C4—C13	1.499 (2)	C18—C19	1.388 (2)
C5—C14	1.475 (2)	C19—C20	1.388 (3)
C5—C6	1.525 (2)	C19—H19	0.9300
C6—C7	1.530 (2)	C20—C21	1.362 (3)
C6—H6	0.9800	C20—H20	0.9300
C7—C8	1.381 (2)	C21—H21	0.9300
C7—C12	1.383 (2)	C22—H22A	0.9600
C8—C9	1.384 (3)	C22—H22B	0.9600
C8—H8	0.9300	C22—H22C	0.9600
C9—C10	1.371 (3)		
			
C2—S1—C3	91.30 (7)	C7—C12—C11	120.65 (16)
C3—N1—C1	116.64 (12)	C7—C12—H12	119.7
C3—N1—C6	119.93 (12)	C11—C12—H12	119.7
C1—N1—C6	122.05 (12)	C4—C13—H13A	109.5
C3—N2—C4	116.54 (13)	C4—C13—H13B	109.5
C21—N3—C18	108.92 (15)	H13A—C13—H13B	109.5
C21—N3—C22	124.10 (15)	C4—C13—H13C	109.5
C18—N3—C22	126.96 (14)	H13A—C13—H13C	109.5
C14—O3—C15	116.02 (13)	H13B—C13—H13C	109.5
O1—C1—N1	123.23 (14)	O2—C14—O3	121.64 (15)
O1—C1—C2	127.04 (14)	O2—C14—C5	126.98 (16)
N1—C1—C2	109.69 (12)	O3—C14—C5	111.37 (13)
C17—C2—C1	122.10 (14)	O3—C15—C16	109.13 (16)
C17—C2—S1	126.93 (13)	O3—C15—H15A	109.9
C1—C2—S1	110.87 (10)	C16—C15—H15A	109.9
N2—C3—N1	126.75 (14)	O3—C15—H15B	109.9
N2—C3—S1	121.76 (12)	C16—C15—H15B	109.9
N1—C3—S1	111.48 (10)	H15A—C15—H15B	108.3
C5—C4—N2	122.14 (13)	C15—C16—H16A	109.5
C5—C4—C13	126.79 (15)	C15—C16—H16B	109.5
N2—C4—C13	111.07 (14)	H16A—C16—H16B	109.5
C4—C5—C14	122.06 (14)	C15—C16—H16C	109.5
C4—C5—C6	120.85 (13)	H16A—C16—H16C	109.5
C14—C5—C6	117.05 (13)	H16B—C16—H16C	109.5
N1—C6—C5	107.91 (11)	C2—C17—C18	128.30 (15)
N1—C6—C7	110.27 (12)	C2—C17—H17	115.8
C5—C6—C7	111.47 (11)	C18—C17—H17	115.8
N1—C6—H6	109.0	N3—C18—C19	106.40 (14)
C5—C6—H6	109.0	N3—C18—C17	121.26 (15)
C7—C6—H6	109.0	C19—C18—C17	132.26 (15)
C8—C7—C12	118.85 (15)	C18—C19—C20	108.23 (17)
C8—C7—C6	119.96 (15)	C18—C19—H19	125.9
C12—C7—C6	121.13 (13)	C20—C19—H19	125.9
C7—C8—C9	120.06 (18)	C21—C20—C19	107.12 (17)
C7—C8—H8	120.0	C21—C20—H20	126.4
C9—C8—H8	120.0	C19—C20—H20	126.4
C10—C9—C8	120.59 (18)	N3—C21—C20	109.33 (16)
C10—C9—H9	119.7	N3—C21—H21	125.3
C8—C9—H9	119.7	C20—C21—H21	125.3
C11—C10—C9	119.81 (17)	N3—C22—H22A	109.5
C11—C10—H10	120.1	N3—C22—H22B	109.5
C9—C10—H10	120.1	H22A—C22—H22B	109.5
C10—C11—C12	120.04 (19)	N3—C22—H22C	109.5
C10—C11—H11	120.0	H22A—C22—H22C	109.5
C12—C11—H11	120.0	H22B—C22—H22C	109.5
			
C3—N1—C1—O1	−178.40 (14)	C5—C6—C7—C8	−101.17 (16)
C6—N1—C1—O1	−11.8 (2)	N1—C6—C7—C12	−43.80 (18)
C3—N1—C1—C2	−0.46 (18)	C5—C6—C7—C12	76.03 (17)
C6—N1—C1—C2	166.11 (12)	C12—C7—C8—C9	−0.6 (2)
O1—C1—C2—C17	2.4 (2)	C6—C7—C8—C9	176.68 (15)
N1—C1—C2—C17	−175.41 (14)	C7—C8—C9—C10	0.9 (3)
O1—C1—C2—S1	179.05 (14)	C8—C9—C10—C11	−0.3 (3)
N1—C1—C2—S1	1.22 (15)	C9—C10—C11—C12	−0.6 (3)
C3—S1—C2—C17	175.15 (15)	C8—C7—C12—C11	−0.3 (2)
C3—S1—C2—C1	−1.27 (11)	C6—C7—C12—C11	−177.55 (14)
C4—N2—C3—N1	5.9 (2)	C10—C11—C12—C7	0.9 (3)
C4—N2—C3—S1	−173.22 (11)	C15—O3—C14—O2	0.3 (2)
C1—N1—C3—N2	−179.70 (15)	C15—O3—C14—C5	179.36 (14)
C6—N1—C3—N2	13.4 (2)	C4—C5—C14—O2	2.3 (3)
C1—N1—C3—S1	−0.50 (16)	C6—C5—C14—O2	−175.46 (18)
C6—N1—C3—S1	−167.38 (10)	C4—C5—C14—O3	−176.79 (14)
C2—S1—C3—N2	−179.73 (14)	C6—C5—C14—O3	5.49 (19)
C2—S1—C3—N1	1.03 (11)	C14—O3—C15—C16	−170.47 (19)
C3—N2—C4—C5	−8.6 (2)	C1—C2—C17—C18	172.86 (15)
C3—N2—C4—C13	170.92 (14)	S1—C2—C17—C18	−3.2 (3)
N2—C4—C5—C14	174.67 (14)	C21—N3—C18—C19	−0.08 (18)
C13—C4—C5—C14	−4.7 (2)	C22—N3—C18—C19	178.08 (16)
N2—C4—C5—C6	−7.7 (2)	C21—N3—C18—C17	177.00 (14)
C13—C4—C5—C6	172.92 (15)	C22—N3—C18—C17	−4.8 (2)
C3—N1—C6—C5	−26.08 (17)	C2—C17—C18—N3	−176.53 (15)
C1—N1—C6—C5	167.78 (13)	C2—C17—C18—C19	−0.3 (3)
C3—N1—C6—C7	95.89 (15)	N3—C18—C19—C20	0.3 (2)
C1—N1—C6—C7	−70.25 (17)	C17—C18—C19—C20	−176.32 (17)
C4—C5—C6—N1	23.55 (19)	C18—C19—C20—C21	−0.4 (2)
C14—C5—C6—N1	−158.71 (12)	C18—N3—C21—C20	−0.2 (2)
C4—C5—C6—C7	−97.67 (16)	C22—N3—C21—C20	−178.41 (17)
C14—C5—C6—C7	80.07 (16)	C19—C20—C21—N3	0.4 (2)
N1—C6—C7—C8	139.00 (14)		
